# Open Science Is Liberating and Can Foster Creativity

**DOI:** 10.1177/1745691618767878

**Published:** 2018-07-02

**Authors:** Willem E. Frankenhuis, Daniel Nettle

**Affiliations:** 1 Behavioural Science Institute, Radboud University; 2 Centre for Behaviour and Evolution and Institute of Neuroscience, Newcastle University

**Keywords:** open science, preregistration, uncertainty, doubt, creativity

## Abstract

Some scholars think that Open Science practices constrain researchers in ways that reduce their creativity, arguing, for instance, that preregistration discourages data exploration and so stifles discovery. In this article, we argue the opposite: Open Science practices are liberating and can foster creativity. Open Science practices are liberating because they (a) enable us to explore data transparently and comfortably; (b) reward quality, which is under our control, rather than outcomes, which are not; and (c) reduce the choke hold of needing to find “positive” results for career advancement. Open Science practices can foster creativity because they cultivate an open and flexible mind-set, create a more collaborative and constructive climate, and generate more accurate information and make it more accessible. In sum, Open Science liberates researchers more than it constrains them.

Those with any imagination and understanding are filled with doubt and indecision.

—Bertrand Russell (1951, pp. 4–5)

I think it’s much more interesting to live not knowing than to have answers which might be wrong. I have approximate answers and possible beliefs and different degrees of certainty about different things. But I’m not absolutely sure of anything, and there are many things I don’t know anything about.

—Richard Feynman (1999, pp. 24–25)

The essence of science is doubt. In fact, science has been described as a system of organized skepticism (Lenoir, 1997). In other belief systems, the ideas held to be true are hardened into dogma, declared as absolute and certain. In science, they should be held provisionally, questioned, continually refined, and replaced. In dogmatic belief systems, epistemic effort is directed toward confirmatory instances: “See, I went to the sacred waterfall yesterday, and today my cold is better.” In science, by contrast, all our epistemic effort should be directed to the anomalies, the cases where the prediction is not met, the instances where the theory breaks down, the puzzling inconsistencies that help reject incorrect claims or stimulate the development of an original idea or new paradigm.

Yet there is a paradox in the scientific status quo: Researchers are incentivized, even encouraged, to deemphasize doubt and uncertainty. Articles are deemed unpublishable, especially by “high-status” journals, if they present findings without being able yet to explain them, if they have multiple experiments with differing results, and if they have mainly null findings (Ferguson & Heene, 2012; Schimmack, 2012).

Scientists are only human. They respond to these incentives. They may present analyses that were really exploratory as if they were confirmatory. They may rehypothesize after the results are known (Kerr, 1998) to give a greater sense of coherence that will satisfy their own confirmation biases, impress fellow scientists, and please journal editors. They selectively present analyses that maximize the impression that results are “significant” and suppress evidence that seems “mixed” (Bakker, van Dijk, & Wicherts, 2012; Button et al., 2013; Chambers, 2017; Ioannidis, 2005; Nosek et al., 2015; Nosek, Spies, & Motyl, 2012; Simmons, Nelson, & Simonsohn, 2011; Vul, Harris, Winkielman, & Pashler, 2009). Given these quite understandable tendencies, it should be no surprise that when studies in the behavioral and life sciences are replicated, we do not consistently see in the new results the patterns reported in the original studies (Begley & Ioannidis, 2015; Camerer et al., 2016; Frank et al., 2017; Klein et al., 2014; Open Science Collaboration, 2015). Thus, we have a paradox. To thrive in a knowledge system based on doubt, it is necessary to downplay doubt and anomaly. In a system in which researchers are free to selectively present analyses, to keep raw data private, and to hypothesize after the facts are known, they feel constrained by the fear of rejection for publication, and its negative career consequences.

## The Open Science Agenda

Fortunately, times are changing. A proliferation of initiatives, collectively known as the Open Science movement, aims to improve science by changing its incentive structure. For example, Open Science argues for the publication of all good-quality data regardless of whether results meet arbitrary significance thresholds (Chambers, 2013, 2017; Nosek & Lakens, 2014; Wagenmakers, Wetzels, Borsboom, van der Maas, & Kievit, 2012), that replication studies should be published (Brandt et al., 2014; Koole & Lakens, 2012; Zwaan, Etz, Lucas, & Donnellan, 2017), that hypotheses and methods should, where possible, be registered before the results are known, that analysis strategies should be transparent and open, and that raw data should be freely available for examination by the community (Morey et al., 2016; Wicherts & Bakker, 2012; Wicherts, Borsboom, Kats, & Molenaar, 2006).

In this article, we focus on the benefits of Open Science for individual scientists. There are many such benefits, but we highlight one: We argue that the new trends in research practices have the potential to liberate researchers and foster their creativity. If we are right, then the field is heading in a great direction: The reliability of science, our personal well-being, and our individual and collective creativity may all be enhanced by a culture that licenses us to be more open, exploratory, uncertain, and transparent. Note also that we focus on Open Science practices, rather than on replication. Although Open Science practices facilitate replication, replication and Open Science are separable. It is quite possible to be fully transparent (e.g., preregistration, open materials and data, transparent reporting, open access publishing) even for studies that cannot be precisely replicated (e.g., because societies have gone extinct or have changed; Greenfield, 2017). Transparency in studies that cannot be replicated is at least as important as in studies that can be replicated and for the same reasons (e.g., knowing how many analyses have been conducted improves estimates of evidentiary strength). Indeed, transparency in nonreplicable studies is arguably even more important: If we can have only one shot at the study, we should invest heavily in maximizing its information value, which increases with greater transparency.

Many of the arguments that have been made for Open Science focus on increasing the reliability of scientific knowledge. In effect, it seems as if researchers are being forced to accept greater constraints on their actions, for the societal good of producing information that is more likely to be true. It is true that Open Science imposes extra constraints, and those extra constraints can feel stifling. Susan Goldin-Meadow (2016), the president of the Association for Psychological Science, is concerned that preregistration will discourage exploration and so obstruct discovery: “[I] fear that preregistration will stifle discovery. Science isn’t just about testing hypotheses—it’s also about discovering hypotheses. . . . Aren’t we supposed to let the data guide us in our exploration? How can we make new discoveries if our studies need to be catalogued before they are run?” (para. 4). In a similar vein, neuroscientist Sophie Scott (2013) notes in a *Times Higher Education* article titled “Pre-Registration Would Put Science in Chains” that she was “also very uncomfortable with the model’s implication that hypothesis testing is the only correct way of doing science. . . . We must be allowed to run studies in which we get things wrong, change our minds and are led in directions we didn’t expect” (para. 9; see also Gonzales & Cunningham, 2015). These thoughts are understandable. However, they are based on a rather zero-sum view: The status quo allows high researcher creativity and freedom but low reliability, whereas Open Science offers greater reliability at the cost of curtailed creativity and freedom. We have a different view: If implemented right, Open Science can be liberating.

## How Open Science Can Be Liberating

The claim that open science is liberating might seem puzzling at first. Consider preregistration: providing a detailed study plan that clearly states, among other things, which statistical analyses are confirmatory and which are exploratory (Nosek, Ebersole, DeHaven, & Mellor, 2018; Wagenmakers et al., 2012). The goal of preregistration is to reduce degrees of freedom in researcher decisions as they collect, process, and analyze data (alongside other goals, such as encouraging more thinking up-front and increasing the efficiency of research designs). Is it not paradoxical to propose that such constraints are liberating?

In truth, however, preregistration *does* allow researchers to explore their data in any way they like, as long as it is clear that is what they are doing. The descriptive and exploratory phases of science are as important as the confirmatory ones, and they should be able to be presented as such. Preregistration also allows researchers to change their research plans (e.g., if one learns about a better statistical analysis), as long as these changes are well motivated and transparently communicated. This is true even for registered reports—a publication format in which peer review of planned studies occurs before the research is conducted, and potential acceptance for publication does not depend on study outcomes (Chambers, 2013; Nosek & Lakens, 2014; van’t, Veer, & Giner-Sorolla, 2016)—in open and honest dialogue with the editors and reviewers. As Stahl and Pickles (2018) note, “Departures from the prespecification can, and often should be made. But when, how and the reasons why should be explicit in the study publication” (p. 1086) In a similar vein, Nelson, Simmons, and Simonsohn (2017) argue that preregistration does not tie researchers’ hands but merely uncovers readers’ eyes. The more transparent and complete the description of the exploratory process is (rather than hidden under the rug or masquerading as confirmatory analyses), the better readers will be able to assign evidentiary value to exploratory findings (Wigboldus & Dotsch, 2016). Even results found in exploration without bookkeeping may be reported, if thus labeled and interpreted with appropriate caution. In short, preregistration does not restrict exploration.

In practice, however, tension might arise when researchers are not able to specify certain aspects of their studies in advance or are forced to deviate from their original plans—for instance, while conducting cross-cultural or field research. Is preregistration even possible, let alone useful, in settings characterized by many free, uncontrollable parameters? Yes. In the design phase, researchers can anticipate some decisions they will have to make (e.g., what age groups to include), and they can create decision trees for contingencies they expect to face (e.g., if-then rules; Nosek et al., 2018). Then, when researchers encounter unexpected setbacks or, for that matter, opportunities (e.g., testing conditions are different from what was anticipated or change during testing), they can take notes of decisions they have to make “on the fly,” as many already do, and report these decisions in manuscripts or supplemental materials.

It is of paramount importance that such transparency in planning and reporting is then rewarded by editors and reviewers, grant panels, and hiring committees. These evaluators must be sensitive to the myriad challenges faced by researchers working in hard-to-control settings. If they are not, the new practices could crucially disadvantage these valuable areas of psychological science. This problem is not entirely new, of course. In the present discourse, too, cross-cultural or field researchers face special challenges in the review process (e.g., inevitably working with small sample sizes, replication studies taking 4 years instead of 4 weeks). If, however, evaluators are appropriately sensitive to these challenges and reward efforts to deal with them soundly, transparent planning and reporting can benefit cross-cultural or field research just as it does experimental research, and for the same reasons. So, we believe that preregistration can benefit all subfields of psychology. But can it really be psychologically liberating? That sounds too good to be true.

For one, preregistration allows us to explore data and change study plans in a tidy and transparent way, without the uncomfortable sense of engaging in illicit activity. It resolves the internal conflict we feel while navigating the garden of forking paths, where we make decisions about our data, after having seen our data (Gelman & Loken, 2014). These feelings of unease have likely increased in the past few years, as we have come to realize that in terms of damage to knowledge, as some have put it, *p* hacking is less like jaywalking and more like drunk driving. With preregistration, we specify how we plan to collect, process, and analyze data before we have seen the data. Moreover, we may start preregistering at an intermediate step (e.g., when working with an existing data set; Nosek et al., 2018). Because verbal descriptions are often ambiguous, some scholars even preregister code for their data analyses. From hearsay and our own experience, we think that scholars find it relaxing not to have to make these critical decisions after having seen the data, accompanied by a lingering sense of guilt, while cognizant of some of their biases and frustratingly unaware of others. How pleasant to be able to assign proper evidentiary value to results, to maximize your chances of obtaining accurate answers to questions that fascinate you, that make your heart thump, and that moved you into science in the first place. And, in the new culture, your colleagues are likely to appreciate these efforts. They might reward your scholarship with professional benefits, such as publications, tenure, or awards. This brings us to the second reason why Open Science practices are liberating: They reward quality, which is under the researcher’s control, rather than outcomes, which are not (Hagen, 2017).

Journals are refocusing their priorities on the quality of research rather than its outcomes (Chambers, 2017); for instance, an increasing number of journals publish registered reports (for an up-to-date list, see https://osf.io/8mpji/wiki/home/). This focus on quality liberates researchers from craving “positive” results (e.g., a significant *p* value) for the purpose of being published. Sure enough, if one’s career depends on publishing in high-impact venues, and these venues favor polished narratives with impeccable results (Fanelli, 2010), then that is what researchers are motivated to provide. Researchers must be allowed to describe research as it is in reality, without having to pretend and paint a seemingly beautiful but less accurate picture, consisting of only significant results.

The alternative is to welcome null results and mixed evidence, as long as the research is well conducted. Indeed, mixed evidence is likely even when there is a true effect (Francis, 2014; Lakens & Etz, 2017; Schimmack, 2012). When journals respect mixed evidence and null results, it reduces the appeal of a small *p* value, which is quite liberating (and, of course, improves the accuracy of the scientific record). That said, scientists may desire “positive” results for other reasons, such as favoring one theory over another or preferring consistency in their own findings. Better practices do not solve all of the world’s problems, just most of them.

The new trends are also liberating for a third reason: They encourage a pluralistic approach to statistics, rather than sole reliance on *p* values. Null-hypothesis significance testing, as it is used in psychological research, forces an all-or-none decision in confirmatory analyses: If the *p* value is smaller than some threshold (e.g., .05), reject the null hypothesis; otherwise, do not reject it. However, evidentiary strength is a matter of degree. Arbitrary thresholds constrain the information value of data. They also invite a false sense of certainty by imposing discrete or even binary conclusions on smooth data (“significant or not?”), with real-life consequences. Do these traffic signs reduce accidents? Does this intervention reduce violence? Does that treatment reduce stress? Treating *p* values as continuous indices emphasizes that inferences do not suddenly assume the mantle of reality (Amrhein & Greenland, 2017; Oaks, 1986; Rosnow & Rosenthal, 1989).

Regardless of *p* values, we often learn more from our data if we compare several competing hypotheses, instead of testing only a single null hypothesis. Therefore, there has been a surge in the use of statistical techniques, such as Bayesian analyses (Gelman et al., 2014; Lee & Wagenmakers, 2014; McElreath, 2015) or model selection and model averaging (Symonds & Moussalli, 2010), that allow us to quantify the relative degrees of support for different hypotheses. These techniques free us from myopic fixation on the null hypothesis, providing insight into a set of hypotheses. They might also foster an open and flexible mind-set capable of entertaining multiple hypotheses simultaneously and pitting them directly against one another, rather than each against the straw man of the null hypothesis. All the while, we update the degree of support for each hypothesis as new evidence comes in. Such an open and flexible mind-set, as we argue in the next section, may contribute to creativity in research by encouraging the exploration of information and its integration into the development of novel and useful solutions.

There is one important caveat we need to add: Open Science requires readers to change just as much as it requires authors to change. If authors embrace doubt and openness, but readers (especially journal editors and peer reviewers) continue to reach for “reject” if papers contain null findings or unplanned exploration, then the already-difficult business of being a scientist becomes even more difficult. Susan Goldin-Meadow would be right that Open Science would be stifling for the individual researcher. But we see Open Science as more than a series of restrictions on individual researchers. Instead, it is an agenda for *systemic* reform, extending to the types of journals that exist (including new types of journals that focus on reform, such as *Meta-Psychology*, the *Journal of Open Psychological Data*, and *Advances in Methods and Practices in Psychological Science*), active use of preprint servers, changing peer-review norms, and better statistical training (Asendorpf et al., 2013; Carlsson et al., 2017; Munafò et al., 2017; Shrout & Rodgers, 2018).

## Open Science Can Foster Creativity

Creativity is the process of generating, selecting, and implementing *novel* and *useful* solutions to problems (Amabile, 1996). In science, a problem is a question. The solution to a question is knowledge. To be novel, knowledge must be unlikely to be generated, selected, or implemented on the basis of the existing ideas and methods. To be useful, knowledge must advance theory or resolve an applied challenge.

The question of whether Open Science fosters creativity can be considered at the level of the individual researcher and at the level of the scientific community as a whole. At the level of the individual researcher, it is true that Open Science practices impose constraints on what scientists can do. We would argue, though, that there are already constraints (e.g., implicit criteria used by reviewers and editors). A great virtue of Open Science is that it transparently reveals these constraints. This is an improvement on the current system, in which constraints are often murky and arbitrary. If all parties know what the constraints are and their application can be checked (e.g., through open peer review), then there is less reason to be anxious and less scope for arbitrary power. If constraints reward transparency, there is every reason for honesty.

Moreover, the idea that any constraint hinders creativity is an old-fashioned one. Contemporary research shows that creativity can be at its highest when there is some degree of constraint, be it of time, budget, or process. For instance, Rosso (2014) finds that R&D teams can benefit creatively from the right kinds of constraints. He notes that his research challenges “the assumption that constraints kill creativity, demonstrating instead that for teams able to accept and embrace them, there is freedom in constraint” (p. 551). The existence of the constraints forces individuals to find novel ways of satisfying them (e.g., reviewers may invite authors to consider questionnaires with more desirable psychometric properties than the ones they are planning to use). With no constraints and unlimited license, it is all too easy to fall back on familiar preexisting beliefs or solutions (e.g., the measurement instruments one has used since graduate school or that are most ubiquitously used in one’s field of study). Clearly, constraints must not be too stringent—exploratory analyses must be encouraged, for example—but their complete absence is neither possible nor desirable.

There should also not be “too many” constraints. Some colleagues are concerned about a bureaucratization of the research enterprise. The time and effort people spend on increasing research transparency does trade off with other activities, including in some cases activities that might be more creative. And some people may not have the patience for doing the additional work needed to increase transparency (e.g., preregister, create data files that other people can understand, share data and materials). They might leave science or forego entering it, resulting in a loss of human capital. Do these potential costs exceed the benefits of Open Science? Although the answer to this question is at least in part empirical (e.g., how many people leave or do not enter, their attributes, the extent to which the new practices actually accelerate the progression of knowledge), we suspect that the vast majority of scholars, including highly creative ones, will come to experience that the personal benefits of Open Science exceed its personal costs. It is less a matter of extra work and more a matter of working in a different way than we have become used to.

We have already noted that a pluralistic approach to statistics may encourage the development of an open and flexible mind-set by inviting researchers to consider multiple hypotheses (rather than only the null hypothesis), while updating degrees of support for each (instead of forcing an all-or-none decision) as new evidence is sampled. However, there are other reasons why the new practices might cultivate an open and flexible mind-set: They change the ecology of science.

Once mixed evidence and modest narratives become common, we habituate to them. We come to expect them. If students read about doubt and uncertainty in textbooks and articles, they learn to assign degrees of evidence to ideas and findings. If scholars can express doubt and uncertainty in their publications (without being rejected), they become less wedded to their ideas and findings, and their reputations less tied to them. This will allow scholars to be more open-minded, less hindered by confirmation bias. If registered reports or preprints allow us to improve *before* we conduct to our studies, why would we stick to suboptimal research designs if colleagues provide useful feedback? Instead of a culture in which scholars are forced to tenaciously defend polished end products, Open Science practices provide platforms for constructive discussion (e.g., clarification of concepts and ideas, measurement of variables, the best statistical analyses), where all parties share the goal of improving a project rather than defending it or judging its suitability for publication. In this more collaborative setting, both authors and reviewers can feel more free to honestly express their doubt and uncertainty. In an open exchange, researchers may be more willing and able to explore unfamiliar terrain and to consider ways of improving weaknesses and enhancing strengths in their work, leading them to discover novel and potentially useful solutions, thus paving the way for creativity (Sternberg, 2006). As reflecting on past failures can improve subsequent performance (DiMenichi & Richmond, 2015), open exchanges could even improve the quality of future projects.

Open Science research practices might also foster the creativity of the scientific community as a whole. Science is a community-level process. Even if individual positions are wrong, or overstated, the scientific community has particular norms and institutions for counteracting this and finding what is useful: peer review of manuscripts, critical review articles, commentaries, replications, meta-analyses. The objectivity of science is not contained within the heads of the individual scientists who come up with the ideas, but rather is distributed across the community of people who review, argue, test, critique, revise, evaluate, and teach. Thus, we need to understand the conditions in which the collective creativity of science will be maximized. This is a multifactorial problem; to address it, scholars have recently turned to experimental studies (Balietti, Goldstone, & Helbing, 2016; Derex, Godelle, & Raymond, 2014) as well as formal models of the scientific process (Bergstrom, Foster, & Song, 2016; Grimes, Bauch, & Ioannidis, 2018; Higginson & Munafò, 2016; McElreath & Smaldino, 2015; Nissen, Magidson, Gross, & Bergstrom, 2016; Smaldino & McElreath, 2016; Zollman, 2010). The results are not obvious a priori. For example, formal modeling suggests that increased connectivity within populations (which leads to more transmission of information) is bad for innovation because people copy the slightly better solutions of others, preventing them from going down a completely novel path (Derex & Boyd, 2016). Our thoughts in this section are therefore particularly tentative and may be overturned by future results.

The new research practices are revolutionizing the ways in which information is generated, selected, and transmitted. Transparent reporting and preregistration are likely to increase the amount and accuracy of the available information. Likewise, increases in open data and open-access publishing raise the probability that other scientists can detect mistakes (e.g., when replicating reported analyses), gain more insight into the data (e.g., by conducting additional analyses), or learn from integrating different data sets (e.g., enabling the analysis of new relationships; Nosek & Bar-Anan, 2012; Nosek et al., 2015; Wicherts et al., 2006). Furthermore, open data will likely to encourage better data management (e.g., labeling and describing variables, storing of data files in repositories), making information less likely to be lost.

With more accurate information being more accessible, we think that science can progress faster toward novel and useful solutions to theoretical questions and applied challenges for at least two reasons. Creative scholars are more likely to look further when they stand on the shoulders of more accurate prior knowledge and they are more likely to look in the right direction when they know which lands have been fruitlessly explored by others (this requires reports of those explorations, regardless of the results). The process of selecting and combining the right bits of information becomes all the more significant (Spellman, 2012). And so does awareness of the bits that do not yet make sense. The failures of our current understanding: the anomalies, the failed predictions, the problems, the things that do not yet fit. That is where the scientific action is. That is where creativity happens.

## Conclusions and Future Direction

We have argued that Open Science liberates researchers and can foster their creativity. The new research practices are liberating because they (a) enable us to explore data transparently and comfortably; (b) reward quality, which is under our control, rather than outcomes, which are not; and (c) reduce the choke hold of needing to find “positive” results for career advancement. The new practices can foster creativity because they cultivate an open and flexible mind-set, create a more collaborative and constructive climate, and generate more accurate information and make it more accessible. We do not think that reliability versus creativity is a zero-sum game: It is possible for knowledge to become more reliable with researchers continuing to enjoy great creativity. This is because Open Science liberates researchers in some ways even as it constrains them.

To end, we highlight one future direction. We have focused our discussion on transparency in empirical studies. It is an interesting question whether other types of research, such as theoretical modeling, can also benefit from increased transparency (we thank the Editor of *Perspectives in Psychological Science*, Robert Sternberg, and Leonid Tiokhin in personal communication, for raising this question). We think they can. In evolutionary psychology, for instance, researchers might disagree about the natural selection pressures that have shaped aspects of human cognition and behavior. A rigorous method for studying the logic and plausibility of evolutionary explanations is to build a mathematical model that formalizes assumptions about the environment (its statistical properties) and organisms (their initial attributes) and computes the expected outcomes of evolution (Frankenhuis, Panchanathan, & Barrett, 2013; Frankenhuis & Tiokhin, 2018). Such modeling can benefit from transparency in several ways, some of which are obvious, but others not.

As is already typically done, researchers can publish code and equations with their manuscript, allowing readers to better evaluate and more easily replicate their work. What we have not seen, however, are theoreticians preregistering the assumptions of their model before computing its results. This practice could prevent researchers from fooling themselves in the garden of forking paths by changing assumptions during the modeling in ways that fit their favored explanation. It can also preempt criticism of other scholars who are concerned that theoreticians engage in such practices (e.g., Bowers & Davis, 2012). And it can help modelers who formalize existing ideas to agree up-front with the founders or proponents of these ideas, who themselves might not be modelers, about assumptions. That way, if modeling results cast doubt on the ideas, these founders or proponents do not change their assumptions after seeing results they do not like, creating a moving target. We have focused on evolutionary modeling, but similar arguments apply to other types of modeling (e.g., Bayesian optimality models).

The same logic applies to other types of research, such as meta-analyses and systematic reviews: By agreeing (or disagreeing) up front about which studies to include, what search terms to use, and so on, we are less likely to fall prey to our own, and each other’s, confirmation biases. In the medical and social sciences, it is already considered good practice to register the protocols of meta-analyses and reviews before beginning data extraction, and an online archive, PROSPERO, to lodge and freely view these protocols already exists (https://www.crd.york.ac.uk/prospero/). The Cochrane Collaboration provides the opportunity for protocols to be peer-reviewed before data extraction (see http://methods.cochrane.org/pma/welcome), the equivalent to a registered report for an empirical study. Thus, the evolution toward greater transparency ahead of time is already under way for reviews and syntheses.
